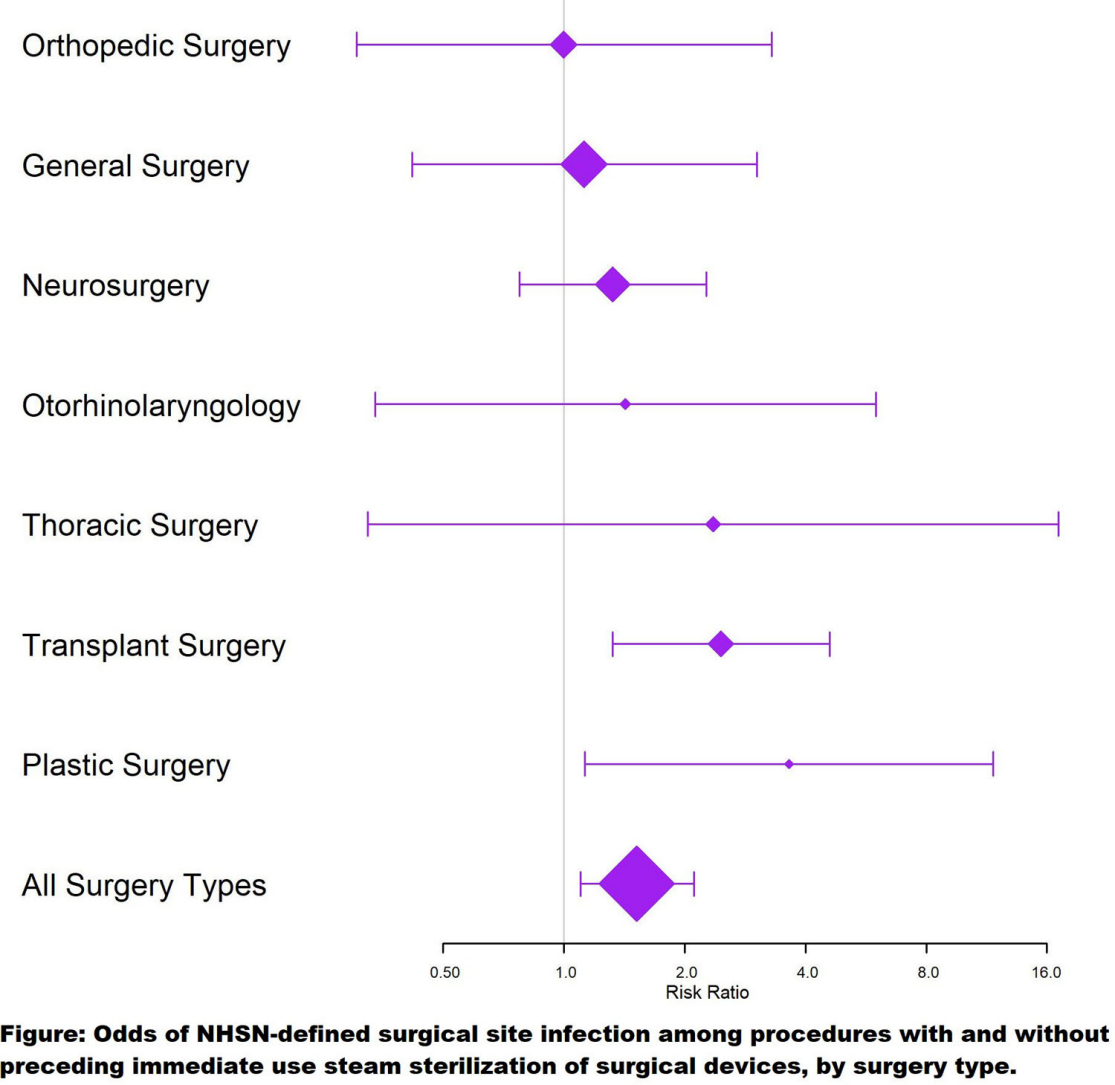# Immediate Use Steam Sterilization and the Effect on Surgical Site Infections in an Acute Care Facility

**DOI:** 10.1017/ash.2024.316

**Published:** 2024-09-16

**Authors:** Casey Lewis, Victoria Crall, Graham Snyder, Ashley Ayres, Avisha Risnear, Jason Miller, Joanne Sherer

**Affiliations:** UPMC; University of Pittsburgh; UPMC Presbyterian

## Abstract

**Background:** Immediate use steam sterilization (IUSS) shortens the time from sterilization to the aseptic transfer onto the surgical sterile field. Published data incompletely defines the extent to which IUSS increases risk of surgical site infection (SSI), compared to standard sterile reprocessing methods. We aimed to measure the association between IUSS use for surgical instrument reprocessing and SSI risk in a facility where IUSS use increased due to staffing constraints and case volumes. **Methods:** In this retrospective observational study at a tertiary care hospital with a diverse mix of surgery types, we used sterile reprocessing logs and SSI outcomes defined using National Health and Safety Network definitions to compare SSI rates among surgeries using surgical devices sterilized using IUSS compared to standard terminal sterilization methods. We calculated a risk ratio (RR) and 95% confidence interval (95%CI), including stratification by eleven high-volume service lines. **Results:** Among 23,919 surgical procedures, 416 (1.74%) developed SSIs. IUSS was used to sterilize instruments prior to 1,524 (6.37%) surgical procedures, and of these procedures 39 (2.56%) developed an SSI, compared to 1.68% of non-IUSS procedures (377 SSI in 22,395 procedures; risk ratio [RR] 1.52, 95% confidence interval [95%CI] 1.10-2.11). Two surgical services had statistically significant RRs for SSI development after IUSS: transplant surgery (RR 2.47, 95%CI 1.32-4.60] and plastic surgery (RR 3.64, 95%CI 1.13-11.74; Figure). **Conclusion:** IUSS is associated with a significant increase in SSIs, including among varied surgery types. IUSS utilization should be minimized.

**Figure:**